# Glucose-Induced Hemodynamic and Metabolic Response of Skeletal Muscle in Heart Failure Patients with Reduced vs. Preserved Ejection Fraction—A Pilot Study

**DOI:** 10.3390/jcdd9120456

**Published:** 2022-12-13

**Authors:** Michael Boschmann, Lars Klug, Frank Edelmann, Anja Sandek, Stephan von Haehling, Hans-Dirk Düngen, Jochen Springer, Stefan D. Anker, Wolfram Doehner, Nadja Jauert

**Affiliations:** 1 Experimental & Clinical Research Center (ECRC), A Joint Cooperation between Charité-Universitätsmedizin Berlin and Max Delbrück Center for Molecular Medicine, 10115 Berlin, Germany; 2 Department of Cardiology, (Virchow Campus) Charité-Universitätsmedizin Berlin, 13353 Berlin, Germany; 3 DZHK (German Centre for Cardiovascular Research), Partner Site Berlin, 10785 Berlin, Germany; 4 Department of Cardiology and Pneumology, Universitätsmedizin Göttingen (UMG), 37075 Göttingen, Germany; 5 German Centre for Cardiovascular Research (DZHK), Partner Site Göttingen, 37075 Göttingen, Germany; 6 Berlin Institute of Health Center for Regenerative Therapies (BCRT), Charité-Universitätsmedizin Berlin, 10117 Berlin, Germany; 7 Division of Physiology, Department of Human Medicine, MSB Medical School Berlin, Rüdesheimerstr 50, 14197 Berlin, Germany

**Keywords:** insulin resistance, skeletal muscle, metabolism, microdialysis

## Abstract

(1) Background: Insulin resistance (IR) is a characteristic pathophysiologic feature in heart failure (HF). We tested the hypothesis that skeletal muscle metabolism is differently impaired in patients with reduced (HFrEF) vs. preserved (HFpEF) ejection fraction. (2) Methods: carbohydrate and lipid metabolism was studied in situ by intramuscular microdialysis in patients with HFrEF (59 ± 14y, NYHA I-III) and HFpEF (65 ± 10y, NYHA I-II) vs. healthy subjects of similar age during the oral glucose load (oGL); (3) Results: There were no difference in fasting serum and interstitial parameters between the groups. Blood and dialysate glucose increased significantly in HFpEF vs. HFrEF and controls upon oGT (both *p* < 0.0001), while insulin increased significantly in HFrEF vs. HFpEF and controls (*p* < 0.0005). Muscle tissue perfusion tended to be lower in HFrEF vs. HFpEF and controls after the oGL (*p* = 0.057). There were no differences in postprandial increases in dialysate lactate and pyruvate. Postprandial dialysate glycerol was higher in HFpEF vs. HFrEF and controls upon oGL (*p* = 0.0016); (4) Conclusion: A pattern of muscle glucose metabolism is distinctly different in patients with HFrEF vs. HFpEF. While postprandial IR was characterized by impaired tissue perfusion and higher compensatory insulin secretion in HFrEF, reduced muscle glucose uptake and a blunted antilipolytic effect of insulin were found in HFpEF.

## 1. Introduction

Heart failure (HF) is complex clinical syndrome and global health burden with an increasing prevalence and growing health care costs [1]. Insulin resistance (IR) is a common feature in HF contributing to symptomatic severity and prognosis [2,3,4]. IR has been implicated in the development of exercise intolerance in HF, as skeletal muscle is a main target of physical exercises [5,6]. Skeletal muscle is a major organ of insulin-stimulated glucose utilization [7]; therefore, development of IR results in impaired glucose metabolism by skeletal muscle in non-diabetic patients with HF. Notably, IR develops intrinsically in patients with HF and independently of the presence of diabetic comorbidity [8]. Pathophysiological mechanism of IR development is complex and not yet clear in its details. Previously, we reported on reduced glucose transporter GLUT4 in skeletal muscle in non-diabetic patients with chronic HF [9]. Further, differences in IR by short insulin sensitivity test (SIST) have been observed between the patients with HF with reduced (HFrEF) and patients with HF with preserved ejection fraction (HFpEF) [10]. However, the corresponding pathophysiological mechanisms of the different metabolic responses on injection of insulin in both HF phenotypes are not known. Different pathways including diminished tissue perfusion (i.e., impaired glucose delivery to muscle cells), impaired insulin signaling, insufficient glucose phosphorylation within the cell, or inappropriate insulin availability might contribute to development of IR [11,12,13]. All these mechanisms affect the skeletal muscle energy metabolism and underlies physical endurance [2].

The technique of Microdialysis allows in vivo investigation of skeletal muscle or adipose tissue metabolism by assessing interstitial fluid metabolites, either at steady state conditions or in dynamic profiles during metabolic loading. This technique has been successfully applied in patients with a wide range of medical conditions, including ischemic stroke [14], chronic HF [15], metabolic syndrome [16] or multiple sclerosis [17]. The aim of the present pilot study was to test the hypothesis, that there are specific differences in the glucose metabolism of skeletal muscle in patients with HFrEF versus HFpEF. We applied the microdialysis technique in order to study the hemodynamic and metabolic response of the *Vastus lateralis* muscle to an oral glucose load (oGL) in patients with HFrEF and HFpEF versus healthy controls.

## 2. Methods

### 2.1. Patients

Male patients with HFrEF and with HFpEF were prospectively studied in this pilot study. All patients were in stable ambulatory condition and on individually adjusted standard medical therapy in accordance with current guidelines (including beta-blockers, angiotensin-converting enzyme inhibitors, mineralocorticoid receptor antagonists, diuretics, and antiplatelet). None of the patients received any kind of antidiabetic therapy. All patients had a disease history for HF of at least 6 months and were in NYHA class I-III. Patients with HFrEF showed clinical symptoms and documented impaired left ventricular ejection fraction ([LVEF] ≤ 45%). Diagnosis of HFpEF was clinically documented and confirmed by transthoracic echocardiography (TTE) according to the current guidelines [18]. Exclusion criteria were presence of medically treated diabetes mellitus type 2, anticoagulant therapy and novel anticoagulants (NOAC), acute decompensated heart failure within last 6 weeks, acute myocarditis or myocardial infarction, acute and chronic inflammatory diseases, immunosuppressive therapy, neurodegenerative disease or acute infection, and a history of cancer shorter than 5 years. Healthy male volunteers of similar age and body mass index served as controls. 

The Institutional Review Board of Charité-Universitätsmedizin Berlin, Germany approved the study protocol (EA2/137/15). The study was conducted in accordance with Good Clinical Practice, and in compliance with the Declaration of Helsinki and applicable European Regulations. Written informed consent was obtained from all participants before study entry. 

### 2.2. Study Protocol

All clinical and metabolic assessments were performed under standardized conditions starting between 8:00 and 9:00 a.m. after an overnight fasting (≥12 h) after a resting period of at least 20 min in supine position in a quiet, air-conditioned room (metabolic ward). From the day before, patients were asked to abstain from (1) any kind of physical activity, (2) beverages containing caffeine and alcohol, and (3) from smoking. 

Fasting insulin resistance was assessed by homeostasis model assessment (HOMA) index as described previously [19]. Briefly, the relative index of insulin resistance (IR, dimensionless or expressed in %) is calculated by the formula:

Insulin resistance = fasting glucose (mmol/L) × fasting insulin (µU/mL)/22.5 (1)

HOMA-IR = 1 corresponds to normal insulin sensitivity, whereas values > 2 indicate the presence of insulin resistance. BMI was calculated as the ratio of weight (kg) and squared height (m^2^).

Hemodynamic and metabolic responses of skeletal muscle were studied by the microdialysis technique at rest and after an oral 75-g glucose load (oGL) given as 300 mL drink (ACCUCHEK^®^ Dextro^®^, O.G.T., Hoffmann-La Roche AG, Grenzach-Wyhlen, Germany). Total time of testing was about 4 h. 

### 2.3. Microdialysis

Skeletal muscle microdialysis was used to monitor interstitial marker metabolites such as glucose for substrate supply, lactate und pyruvate for anaerobic and aerobic glucose metabolism, and glycerol for lipid mobilization [9]. Briefly, a microdialysis probe (M71, µDialysis AB, Stockholm, Sweden) was inserted into the *Vastus lateralis* muscle after local anesthesia with lidocaine. After probe insertion, a 60 min period was allowed for tissue recovery from insertional trauma and for baseline calibration. Microdialysis probes were perfused with lactate-free Ringer’s solution supplemented with 50 mmol/L ethanol at flow rate of 2 µL/min using a high precision microdialysis pump (M107, µDialysis AB, Stockholm, Sweden). Ethanol was added to assess changes in tissue perfusion by using the ethanol dilution technique based on Fick’s principle [9]. Dialysate samples were taken in 15-min intervals over 30 min before (baseline) and 120 min after the oGL. A vein catheter (Vasofix^®^ Safety, 20 G; B. Braun, Melsungen, Germany) was placed into the antecubital vein for repeated blood sampling in order to assess baseline and postprandial blood glucose and insulin levels. Blood samples were taken before and in 15-min intervals within the first and 30-min intervals within the second hour after the oGL. 

### 2.4. Assays

All blood samples were processed immediately in a refrigerated centrifuge at 4.0 °C and aliquots (serum and plasma) were stored at −80 °C until analysis. Blood glucose and insulin as well as routine parameters were measured according to international standards; ethanol in the perfusate and dialysate with a standard spectrophotometric enzymatic assay; dialysate glucose, lactate, pyruvate and glycerol with an automated analyzer based on colorimetric principles (ISCUSflex, µDialysis, Stockholm, Sweden). In situ dialysate recovery for metabolites was about 50%, as assessed by near-equilibrium dialysis.

### 2.5. Clinical Assessments 

#### 2.5.1. Transthoracic Echocardiography

Participants underwent a Doppler echocardiographic evaluation (Vivid S5 with 3S-RS 1.5–3.6 MHz transducer, GE Medical Systems) according to a standardized protocol. Data regarding cardiac morphology, global ventricular function, and diastolic function were recorded by M-mode, two dimensional and Doppler echocardiography. Left ventricular ejection fraction (LVEF) was calculated from end-systolic and end-diastolic volume evaluated from apical four-chamber and two-chamber view according to Simpson’s method. Peak E and A wave velocities and E wave deceleration time were measured from the mitral leaflet tip. Longitudinal time Doppler velocities, early diastolic wave (E’) and atrial wave (A’) were assessed by pulsed wave tissue Doppler imaging in the apical four-chamber view in septal and lateral ventricular wall. Diagnosis of HFpEF was confirmed by echocardiography according to the ASE/EACI Guidelines 2016 [18].

#### 2.5.2. Statistical Analysis

All evaluations were done as descriptive statistics with no correction for multiple testing. Biochemical variables were tested for normal distribution using the Kolmogorov-Smirnov test. All data were presented as mean ± standard deviation (SD), median (interquartile range, IQR) or mean ± standard error of the mean (SEM) for repeated measurements after the oGL. T-test for paired samples, *t*-test for independent samples and analysis of variance (ANOVA) followed by Fisher’s *post hoc* test were used as appropriate. Global fitting was used to compare the response curves for plasma glucose and insulin and skeletal muscle tissue dialysate concentrations of glucose, lactate, pyruvate and glycerol between treatment groups after the oGL. This test is a non-linear regression method in which one curve (healthy controls) is used as reference, allowing evaluations of discrepancy of the other curve (patient groups). All *p*-values given in the Figures refer to global fitting. Non-normally distributed data were log-transformed to allow parametrical analysis. A value of *p* < 0.05 was considered statistically significant. Statistical analysis was performed with the Statview 5.0 (SAS Institute, Cary, NC, USA) or GraphPad Prism (InStat, Version 6.0 Graphpad Software Inc., San Diego, CA, USA) software.

## 3. Results

### 3.1. Clinical Characteristics of Study Population 

Clinical characteristics of the study groups are presented in Table 1. All study groups were of similar age and BMI. There were no significant differences in clinical lab parameters such as glucose, triglycerides, cholesterol, hemoglobin, HbA1c and creatinine between the study groups. However, there were a tendency of higher values for insulin and HOMA-IR in HFrEF and specifically in HFpEF vs. controls. LV end-diastolic diameter and LVEF as well as tricuspid annular plane systolic excursion were more severely impaired in patients with HFrEF compared to HFpEF (Table 2).

### 3.2. Plasma Glucose and Insulin Profiles 

**Figure 1:**
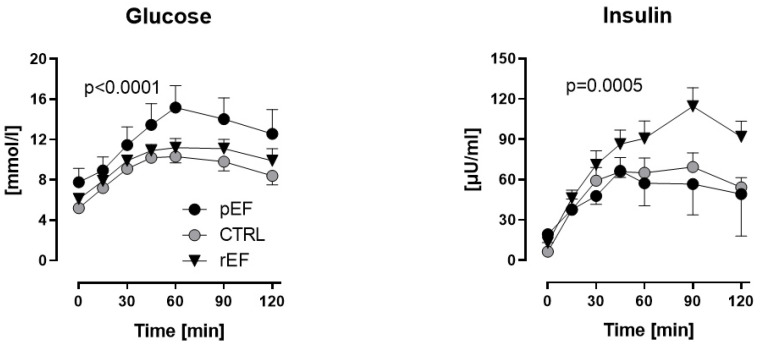
Plasma glucose and insulin levels in patients with heart failure with reduced ejection fraction (rEF, n = 12 men) or patients with preserved ejection fraction (pEF, n = 6 men) and healthy controls (n = 8 men) at baseline and after an oral glucose load (75 g). Data are given as mean ± SE; HFpEF vs. controls, *p* < 0.0001; HFrEF vs. controls, *p* = 0.0005.

There were no significant differences in fasting plasma glucose levels and HOMA-IR between the study groups (Figure 1, Table 1), although HOMA-IR tended to be higher in HFpEF vs. two other groups. Following the oGL, plasma glucose levels raised significantly in all three groups within 60 min, but this increase was significantly higher in HFpEF vs. HFrEF and healthy controls (15.2 ± 2.2 vs. 11.2 ± 0.9 and 10.3 ± 0.6 mmol/L, *p* < 0.0001, respectively; Figure 1, *left panel*). Plasma glucose levels remained elevated (>8 mmol/L) in HFpEF vs. HFrEF and controls even 120 min after the oGL (12.6 ± 2.4 vs. 9.9 ± 1.2 and 8.4 ± 0.9 mmol/L, *p* < 0.0001, respectively; Figure 1, *left panel*).

Fasting plasma insulin levels tended to be higher in HFrEF and HFpEF patients vs. controls (13.0 ± 2.1 and 19.3 ± 6.2 vs. 6.4 ± 0.7 µU/mL, *p* = 0.1, respectively; Figure 1). Plasma insulin levels increased after the oGL in all three groups with the highest increase of insulin levels in HFrEF vs. HFpEF and controls 60 min after the oGL (91 ± 13 vs. 57 ± 17 and 65 ± 11 µmol/mL, *p* < 0.05, respectively). Even 120 min after the oGL, plasma insulin levels remained the highest in HFrEF vs. HFpEF and controls (92 ± 12 vs. 49 ± 32 and 54 ± 7 µmol/mL, *p* = 0.0005, respectively; Figure 1, *right panel*). 

### 3.3. Skeletal Muscle Metabolism

#### 3.3.1. Skeletal Muscle Perfusion and Glucose Uptake 

**Figure 2:**
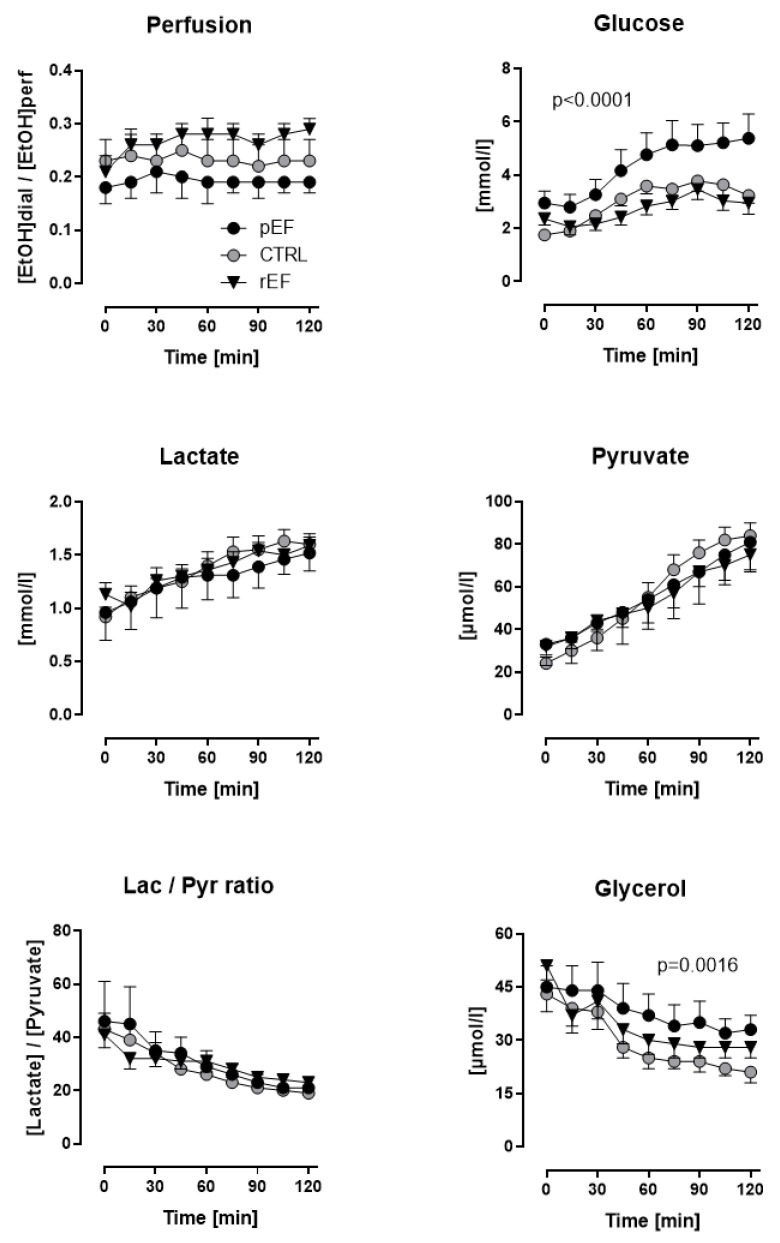
Skeletal muscle (*Vastus lateralis*) ethanol ratio and dialysate concentrations glucose, lactate, pyruvate, and glycerol of patients with heart failure with reduced ejection fraction (rEF, n = 12 men) or patients with preserved ejection fraction (pEF, n = 6 men) and healthy controls (n = 8 men) at baseline and after an oral glucose load (75 g). Data are given as mean ± SE; *upper right panel*: HFpEF vs. controls, *p* < 0.0001; *lower right panel:* HFpEF vs. controls, *p* = 0.0016.

Muscle tissue perfusion as assessed by the ethanol ratio was similar in fasting condition between the patients with HFrEF, HFpEF and controls (0.21 ± 0.03, 0.18 ± 0.03, and 0.23 ± 0.04, n.s., respectively) (Figure 2, *upper left panel*). Whereas this ethanol ratio remained unchanged during the oGT in HFpEF patients and controls, it increased in HFrEF patients vs. baseline (*p* = 0.057) after glucose load, indicating lower postprandial muscle tissue perfusion in HFrEF (Figure 2, *upper left panel*).

Fasting dialysate glucose levels were significantly higher in HFpEF but not HFrEF patients vs. controls (2.95 ± 0.45 vs. 2.36 ± 0.24 and 1.75 ± 0.12 mmol/L, *p* < 0.0001). After the oGL, dialysate glucose increased significantly in HFpEF vs. HFrEF and controls. Sixty minutes after the oGL, dialysate glucose levels achieved 4.77 ± 0.82, 2.83 ± 0.32 and 3.59 ± 0.29 mmol/L in HFpEF, HFrEF and controls, respectively (*p* < 0.0001, Figure 2, *upper right panel*). 

#### 3.3.2. Aerobic and Anaerobic Glycolysis

Fasting dialysate lactate as an indicator of anaerobic glucose utilization (about 1 mmol/L) and pyruvate as an indicator of aerobic glucose utilization (about 30 µmol/L) levels were similar in all three groups (Figure 2, *middle left and middle right panels*). A similar increase of about 1.5-fold in lactate and about 2.5-fold in pyruvate levels was observed 120 min after the oGL in all study groups.

Because of the identical changes in dialysate lactate and pyruvate after the oGL in all three groups, lactate-to-pyruvate ratio decreased similarly by about 50% in all three groups. This indicates a proportional increase in aerobic and anaerobic glycolysis in HFpEF, HFrEF and controls. (Figure 2, *lower left panel*). 

#### 3.3.3. Lipid Mobilization/Lipolytic Activity

Fasting dialysate glycerol levels were similar in all three groups (about 45 µmol/L). Postprandial dialysate glycerol levels, the marker of tissue lipid mobilization, decreased in all study groups Figure 2, *lower right panel*). However, the slope of glycerol levels after oGT was lower in HFpEF and at the end of the test, dialysate glycerol was highest in HFpEF (33 ± 4 µmol/L) vs. controls (21 ± 3 µmol/L, *p* = 0.0016), vs. HFrEF (28 ± 3 µmol/L). This suggests a blunted anti-lipolytic effect of insulin in HFpEF vs. HFrEF and controls.

## 4. Discussion

The main findings of the present study are distinctly different patterns of skeletal muscle insulin resistance (IR) in both HFrEF and HFpEF patients. In HFpEF patients, we found higher postprandial blood and dialysate glucose values at rather normal insulin levels and postprandial dynamics, indicating an impaired glucose uptake not yet compensated by an increased insulin response. A reduced decrease in dialysate glycerol after the oGL also indicates a blunted anti-lipolytic effect of insulin in HFpEF. By contrast, in HFrEF patients, we found postprandial blood and dialysate glucose values within a rather high normal range, at significantly higher postprandial insulin secretion, indicating tissue insulin resistance. The postprandial decrease in dialysate glycerol was similar to the healthy controls, indicating a compensated anti-lipolytic effect upon higher insulin secretion.

In HFrEF patients, elevated blood glucose levels above 8 mmol/L two hours after the oGL and postprandial insulin levels above 100 µU/mL clearly indicate the presence of IR. Lower postprandial muscle tissue perfusion as indicated by an elevated ethanol ratio, suggests a proportionally lower glucose supply into the muscle. However, postprandial dialysate glucose levels are close to the levels of the controls, which could be an indicator of an impaired muscle glucose uptake in patients with HFrEF. In accordance, we observed elevated glucose plasma levels. Impaired hemodynamics secondary to reduced LVEF, reduced peripheral blood flow [20] and peripheral endothelial dysfunction [21,22] may contribute to insufficient organ and tissue perfusion in HFrEF [23,24]. Indeed, we observed lower systemic blood as well as a high-degree reduced LVEF and dilated left ventricle by echocardiography in these patients. At the same time, low postprandial skeletal muscle perfusion may be responsible for truncated delivery of insulin to muscle cells as well. Although the insulin concentrations have not been measured in dialysates in this study, we might assume this by elevated plasma levels of the insulin. Insufficient stimulation of muscle cells by insulin would result in lower expression of the glucose transporter GLUT4 at the cell membrane and in lower glucose uptake [25]. Indeed, previously we have shown a reduced expression of GLU4 in the skeletal muscle [9]. Thus, impaired skeletal muscle tissue perfusion in HFrEF might cause low interstitial glucose and insulin supply that consequently results in scarce energy supply of the muscle and impaired energy metabolic balance, and leads to skeletal muscular weakness and exercise intolerance. 

In patients with HFpEF, elevated HOMA-IR index > 4 under the fasting conditions and elevated postprandial blood glucose levels present perhaps another pathophysiological mechanism of IR as in HFrEF. Notably, fasting plasma insulin levels were the highest in HFpEF, but during the oGL, they have been reduced to values even lower than in the controls. Further, in fasting conditions as well as during the oGL, dialysate glucose levels were elevated in HFpEF suggesting truncated glucose uptake by skeletal muscle cells. In parallel, there was a higher degree of lypolytic activity, indicated by higher dialysate glycerol levels. These findings might suggest an inappropriate insulin production under the oGL in HFpEF. However, insulin fulfilled its peripheral anabolic functions by its canonical phosphokinase PI3K-Akt signaling pathway that has two main effects: (1) it activates glycolysis by activating the glucose transporter 4 (GLUT 4) in the skeletal muscle, and (2) it inhibits postprandial lipolysis, as indicated by a reduction of tissue glycerol—a marker of lipolysis [26]. Both effects seem to be impaired in HFpEF, which clearly supports the presence of IR in HFpEF.

The pathophysiology of HFpEF is still not fully understood. The growing evidence suggests phenotypical differences between the HFrEF and HFpEF regarding the metabolic alterations [27,28]. Our data indicate that the metabolic effects of insulin are impaired in both, HFpEF and HFrEF patients, although at different levels. Interestingly, muscle glucose utilization itself is likely to be intact in patients with HFpEF and HFrEF, as indicated by comparable decreases of the lactate/ pyruvate ratio vs. controls within a physiological range after oGL. This ratio indicates the relation between anaerobic (lactate-production) and aerobic (pyruvate- production) glycolysis. 

Our findings are in line with previous clinical and experimental reports by our group and others. Glucose intolerance is an established risk factor in the development and progression of both HF phenotypes [29]. Previously, a relationship between insulin resistance, higher NYHA, and impaired prognosis has been shown in patients with chronic HF [4]. In addition, differences between HFpEF and HFrEF in insulin resistance in non-diabetic patients who underwent a short insulin sensitivity test have been reported [10]. In this study, patients with HFrEF showed a lower glucose clearance during the in vivo insulin stimulation compared to patients with HFpEF and healthy controls. This might be explained by impaired hemodynamics and lower skeletal muscle perfusion in HFrEF. A pattern similar to the controls of glucose response to insulin stimulation in parallel with elevated plasma glucose levels might indicate insufficient insulin production in HFpEF. 

## 5. Study Limitations

This is a pilot study in a small cohort of patients. However, the number of subjects per subgroup is similar to previous studies using the microdialysis technique for intramuscular in vivo dynamic metabolic assessment and seemed also to be sufficient [14,30]. Furthermore, patients on anticoagulant therapy could not be included into the trial due to elevated bleeding risks from intramuscular placing of the microdialysis probe. It is unlikely, however, that patients on oral anticoagulation may present different characteristics of glucose metabolism. Only male subjects were investigated in this pilot study in order to exclude gender-related confounding variables of metabolic control; therefore, potential sex-dependent differences of the metabolic state cannot be addressed. This will be an issue for upcoming studies. A relation between the muscle functional capacity and insulin resistance in heart failure has been shown previously [31] and was not part of the current study protocol.

## 6. Conclusions

Our study has demonstrated distinctly different patterns of skeletal muscle insulin resistance in patients with HFrEF vs. HFpEF. In HFrEF patients, insulin resistance might be linked to impaired tissue perfusion secondary to hemodynamic failure and to impaired blood glucose transport, while in HFpEF, insulin resistance might be caused by an insufficient insulin cellular signaling. Furthermore, the anti-lipolytic effect of insulin seems to be blunted in HFpEF vs. HFrEF. These findings provide novel insights into distinct metabolic characteristics of HFrEF and HFpEF. It supports the use of antidiabetic agents in patients with HF without diabetes mellitus in order to reduce systemic glucose levels. Better understanding of specific mechanisms of impaired glucose metabolism in separate categories of HF may contribute to a specific therapeutic concept to improve energy utilization in patients with heart failure.

## Figures and Tables

**Table 1:** Baseline clinical characteristics of study groups.

Parameters	Controls n = 8	HFrEF n = 12	HFpEF n = 6	*p*-Value
Age, years	61 ± 10	58 ± 15	64 ± 10	0.6
Body mass index, kg/m^2^	25.5 ± 2.0	26.7 ± 3.5	26.6 ± 5.2	0.8
Systolic blood pressure, mmHg	128 ± 12	110 ± 20 *	132 ± 3 †	0.03
Diastolic blood pressure, mmHg	79 ± 7	67 ± 13 *	79 ± 7 †	0.03
Mean blood pressure, mmHg	96 ± 8	81 ± 15 *	98 ± 10 †	0.03
Glucose, mmol/L	5.3 ± 0.1	7.4 ± 3.1	6.5 ± 2.6	0.5
Insulin, µmol/mL, median (IQR)	4.7 (4.5–5.2)	7.3 (4.3–17.1)	10.2 (7.0–30.0)	0.1
HOMA-IR index, median (IQR)	1.1 (1.1–1.8)	1.8 (1.1–4.8)	4.9 (1.7–9.6)	0.08
Triglycerides, mg/dL	98 ± 25	123 ± 62	92 ± 31	0.4
Cholesterol, mg/dL	190 ± 25	146 ± 23 *	177 ± 60	0.07
High density lipoprotein, mg/dL	57 ± 13	45 ± 18	44 ± 16	0.2
Low density lipoprotein, mg/dL	113 ± 23	76 ± 13 *	105 ± 53	0.08
ASAT, U/L	21.7 ± 3.9	27.0 ± 8.8	26.8 ± 5.9	0.3
ALAT, U/L	23.7 ± 9.7	22.9 ± 12.4	31.2 ± 12.4	0.4
Hemoglobin, g/dL	14.4 ± 1.4	13.6 ± 1.0	12.7 ± 2.3	0.4
HbA1c, %	5.4 ± 0.6	5.5 ± 0.4	6.0 ± 0.9	0.3
Creatinine, mg/dL	0.94 ± 0.14	1.1 ± 0.2	1.0 ± 0.3	0.2
Midregional pro-ANP, nmol/L, median (IQR)	67 (23–85)	385 (145–1184)	177 (159–398)	<0.05
Medication				
Angiotensin-converting enzyme inhibitor, n		9	3	0.3
Angiotensin receptor blocker, n		4	2	1.0
β-blocker, n		10	4	0.4
Diuretics, n		9	4	0.7
Aldosterone-antagonist, n		5	1	0.3
Statins, n		6	3	1.0
Antiplatelet, n		7	4	0.7
Novel oral anticoagulants, n		3	-	

ALAT, alanine aminotransferase; ASAT, aspartate aminotransferase; HOMA-IR, homeostasis model assessment—insulin resistance; IQR, interquartile range. Data are presented as mean ± SE if not otherwise indicated. * *p* < 0.05 vs. controls; † *p* < 0.05 vs. HFrEF.

**Table 2:** Baseline cardiac characteristics of patient groups.

Parameters	HFrEF n = 12	HFpEF n = 6	*p*-Value
NYHA class I/II/III, n [/%]	1.9 ± 0.7	1.3 ± 0.5	0.2
Heart rate, beats per min	65 ± 10	69 ±8	0.9
LV ejection fraction, %	32 ± 10	52 ± 2	0.0003
LV end-diastolic diameter, mm	63.8 ± 12.7	52.6 ± 4.8	0.05
Intraventricular systolic septum thickness, mm	10.5 ± 1.3	11.2 ± 2.6	0.5
LV posterior wall thickness, mm	11.4 ± 1.3	11.0 ± 1.9	0.7
Left atrial diameter, mm	42.0 ± 8.6	46.7 ± 10.8	0.4
Maximal left atrial volume index, mL/m^2^	36.5 ± 6.2	39.7 ± 9.8	0.3
Mitral E-wave velocity, cm/s	62.5 ± 32.2	71.1 ± 14.9	0.7
Mitral A-wave velocity, cm/s	58.2 ± 33.6	63.8 ± 19.8	0.7
Septal e’ mitral annular velocity by TDI, cm/s	5.4 ± 2.6	6.3 ± 1.8	0.5
Lateral e’ mitral annular velocity by TDI, cm/s	7.5 ± 3.1	7.4 ± 1.9	0.6
Mitral E/e’ septal-lateral ratio	11.0 ± 5.5	13.2 ± 2.0	0.1
Aortic valve velocity, m/s	1.2 ± 0.1	1.7 ± 0.9	0.3
Right ventricular end-diastolic parameter, mm	36.4 ± 5.9	34.3 ± 6.8	0.6
Tricuspid annular plane systolic excursion, mm	14.7 ± 5.1	26.0 ± 5.2	0.02

LV, left ventricular; NYHA, New-York Heart Association; TDI, Tissue Doppler Imaging. Data are presented as mean ± SE if not otherwise indicated.

## Data Availability

The data presented in this study are available on request from the corresponding author. The data are not publicity available due to privacy.
